# Response to Galcanezumab in a Patient with Complex Neuropsychiatric Comorbidity: A Case Report

**DOI:** 10.3390/jcm15176540

**Published:** 2026-08-24

**Authors:** Anna Anselmo, Maria Pagano, Francesco Corallo, Irene Cappadona, Rosario Grugno, Domenico Cosenza, Gianluca Vita, Riccardo Lo Presti, Davide Cardile, Viviana Lo Buono, Rocco Salvatore Calabrò

**Affiliations:** IRCCS Centro Neurolesi Bonino-Pulejo, 98124 Messina, Italy; anna.anselmo@irccsme.it (A.A.);

**Keywords:** migraine, galcanezumab, anti-CGRP monoclonal antibodies, central sensitization, psychiatric comorbidity, neuropsychiatric comorbidity

## Abstract

**Background:** Migraine is a leading cause of disability worldwide and is frequently associated with psychiatric comorbidities that may influence pain perception and treatment response. Anti-CGRP monoclonal antibodies represent an effective preventive treatment, although response variability remains poorly understood, particularly in patients with complex neuropsychiatric profiles. **Case Presentation:** We report the case of a 49-year-old woman with migraine with a previously chronic course and psychiatric comorbidity, including bipolar disorder and obsessive–compulsive disorder, treated with galcanezumab. The clinical course was characterized by an “on-off-on” pattern, with marked worsening after treatment discontinuation and subsequent improvement after treatment reintroduction. **Results:** The patient presented with severe emotional distress (BDI-II = 59; STAI = 77–80), a very high self-reported burden of central sensitization-related symptoms (CSI = 100/100) and reduced performance on cognitive screening measures (MoCA = 19/30; BCSE = 18/58). MMPI-3 findings indicated a high level of self-reported psychological distress; however, substantial elevations in symptom-validity indicators required cautious interpretation of the substantive scales. **Conclusions:** This case illustrates that complex neuropsychiatric comorbidity and psychotropic polypharmacotherapy did not preclude an apparent clinical benefit from galcanezumab. Treatment discontinuation and reintroduction were temporally associated with worsening and subsequent improvement in headache frequency, although causality cannot be established from a single uncontrolled observation. The case also highlights the potential value of coordinated neurological and psychiatric monitoring in patients with migraine and complex neuropsychiatric profiles.

## 1. Introduction

Migraine is a highly disabling neurological disorder, particularly in patients with frequent or chronic attacks, and represents a major contributor to disability worldwide. Psychiatric comorbidities are common in individuals with migraine, particularly depressive and anxiety disorders, and may contribute to greater clinical burden, increased migraine-related disability, and greater complexity of disease management [1,2]. The coexistence of psychiatric disorders may also influence prognosis and treatment planning, supporting the need for integrated neurological and psychiatric assessment in clinically complex patients [3].

Monoclonal antibodies targeting the calcitonin gene-related peptide (CGRP) pathway represent an effective and generally well-tolerated preventive treatment option for migraine. Their efficacy has been demonstrated in randomized clinical trials and subsequently confirmed in real-world studies involving more heterogeneous and treatment-resistant populations [4,5]. Nevertheless, treatment response remains variable, and longitudinal real-world evidence suggests that some patients may experience delayed, incomplete, or fluctuating responses, as well as worsening following treatment interruption [5,6].

Clinical management may be particularly challenging in patients with psychiatric comorbidities and concomitant psychotropic polypharmacotherapy. Psychiatric disorders have been associated with greater migraine burden and disability, while evidence specifically addressing anti-CGRP treatment in patients with complex psychiatric profiles remains comparatively limited [3,7]. Real-world observations are therefore valuable for characterizing treatment effectiveness and clinical trajectories in patients whose complexity may not be fully represented in controlled trials [5,8].

In the present case, galcanezumab was introduced only after several previous preventive treatments, including amitriptyline, topiramate, flunarizine, propranolol, and escitalopram, had provided only partial clinical benefit. The subsequent longitudinal course, characterized by treatment discontinuation and reintroduction, provides an opportunity to describe changes in headache frequency within a complex neuropsychiatric and pharmacological context. Importantly, the psychological and cognitive assessment was performed only at a later stage of follow-up and should therefore be regarded as complementary characterization of the patient rather than as an explanation of the observed treatment response.

In this context, the present case aims to describe the longitudinal clinical response to galcanezumab in a patient with complex neuropsychiatric comorbidity and psychotropic polypharmacotherapy, with particular attention to the temporal relationship between treatment discontinuation, reintroduction, and changes in headache frequency. A secondary aim is to characterize the patient’s psychological and cognitive profile at the March 2026 assessment and to discuss its clinical relevance within a multidisciplinary management framework, without inferring a causal relationship with treatment response.

### Case Report

We present the case of a 49-year-old woman who had been followed at our Headache Center since 2018 with a specialist diagnosis of migraine with a previously chronic course. Available clinical records described unilateral headache with side alternation, associated with photophobia, phonophobia, nausea, vomiting, and worsening with physical activity. During subsequent follow-up, the clinical phenotype evolved toward episodic high-frequency migraine without aura. No typical migraine aura was documented in the available headache records. Medication-overuse headache was not documented in the available records. Neurological examination was reported as normal during follow-up visits, and brain MRI with MR angiography had been performed as part of the diagnostic work-up. Although the available records documented a specialist diagnosis of migraine with a previously chronic course, the retrospective records did not consistently report separate monthly headache-day and migraine-day counts; therefore, fulfillment of all individual ICHD-3 criteria for chronic migraine during the earlier disease phase could not be retrospectively verified.

Over the years, the patient underwent several preventive treatments, including amitriptyline, topiramate, flunarizine, propranolol, and escitalopram, with only partial benefit, whereas acute treatment with almotriptan was effective in controlling individual attacks.

In February 2023, preventive treatment with galcanezumab was initiated with a 240 mg loading dose, followed by 120 mg once monthly as maintenance treatment. No major tolerability issues were documented. The patient reported some gastrointestinal disturbances during treatment, although the available clinical records did not establish a clear causal relationship with galcanezumab. Galcanezumab was discontinued between January and February 2024 as part of a planned clinical reassessment. During the first month after discontinuation, the patient reported approximately four attacks per month; thereafter, attack frequency increased to more than seven attacks per month, with greater reported intensity. Galcanezumab was reintroduced in March 2024. The same 120 mg monthly maintenance regimen was resumed. Following treatment resumption, headache frequency decreased initially to approximately 3–4 mild attacks per month and subsequently to 1–2 attacks per month during the following months This clinical course was temporally compatible with renewed benefit following galcanezumab reintroduction, although causality cannot be established from a single uncontrolled clinical observation.

The patient presented with a complex neuropsychiatric profile, including bipolar disorder and obsessive–compulsive disorder with contamination-related themes. The diagnosis of bipolar disorder preceded the onset of migraine. The clinical history also included insomnia, episodes of sleep paralysis, and visual perceptual phenomena described as insect-related hallucinations. No structured assessment of alcohol or substance use was performed as part of the present evaluation. She was receiving psychiatric polypharmacotherapy, including olanzapine, aripiprazole, lamotrigine, paroxetine, and alprazolam. Medical comorbidities included thyroid disease and a pituitary microadenoma. An electroencephalogram performed in December 2025 showed poorly modulated brain electrical activity with bilateral fronto-parieto-temporal paroxysmal abnormalities. A follow-up EEG reassessment was planned within 3–6 months. No causal relationship was inferred between the EEG abnormalities, the headache disorder, and the reported visual perceptual phenomena. Psychometric evaluation revealed a marked affective and anxiety burden, with a Beck Depression Inventory-II (BDI-II) score of 59 and very high State-Trait Anxiety Inventory scores (STAI-Y1 = 77; STAI-Y2 = 80). The Central Sensitization Inventory (CSI) score was 100. From a cognitive perspective, the Montreal Cognitive Assessment (MoCA) score was 19. Treatment adherence was assessed using the 8-item Morisky Medication Adherence Scale (MMAS-8); the obtained score of 1.25 was classified as low adherence according to the adopted scoring criteria. Patient engagement was evaluated using the Patient Health Engagement Scale (PHE-S). These findings provide a cross-sectional characterization of the patient’s psychological and cognitive status at the March 2026 assessment.

**Figure 1:**
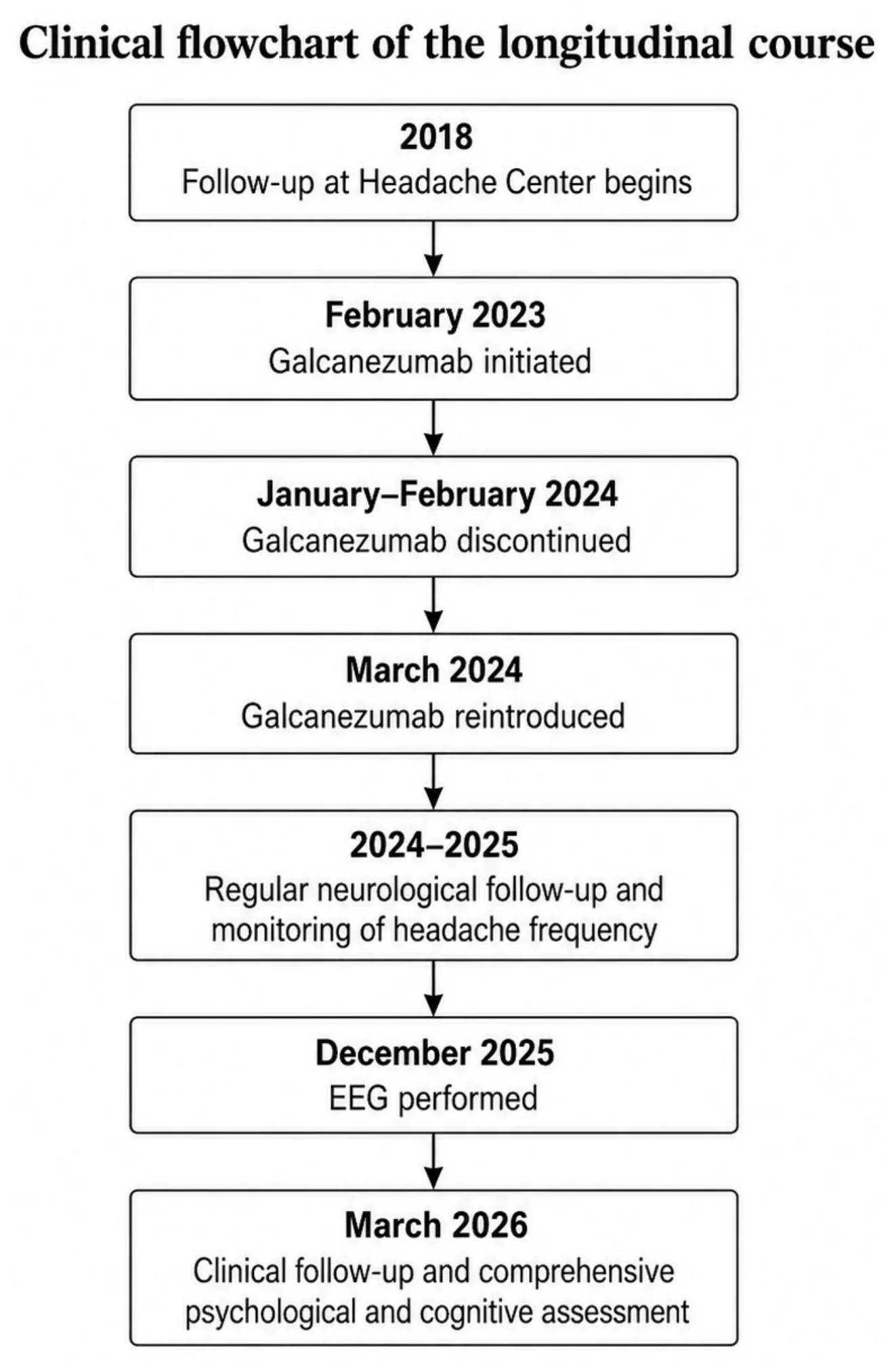
Clinical flowchart of the longitudinal course, including galcanezumab initiation, temporary discontinuation and reintroduction, subsequent neurological follow-up, EEG assessment, and the timing of the comprehensive psychological and cognitive evaluation.

The main clinical milestones, preventive treatment phases, headache frequency, and available headache-related disability measures are summarized in Table 1. A schematic overview of the longitudinal clinical course, including galcanezumab initiation, temporary discontinuation and reintroduction, subsequent neurological follow-up, EEG assessment, and the timing of the comprehensive psychological and cognitive evaluation, is provided in Figure 1.

The increase in HIT-6 score from 48 in September 2024 to 60 in December 2024, despite a similar reported attack frequency, was retained as documented in the clinical records and may reflect differences between attack frequency and the perceived impact of headache during the respective recall periods.

## 2. Materials and Methods

The comprehensive assessment included clinical, neuropsychological, and psychometric measures covering pain intensity, central sensitization-related symptoms, cognitive functioning, personality and psychopathology, patient engagement, medication adherence, and psychological distress. The assessment domains and corresponding instruments are summarized in Table 2.

## 3. Results

### 3.1. Clinical and Psychometric Assessment

At the March 2026 assessment, depressive symptoms were markedly elevated, with a Beck Depression Inventory-II (BDI-II) score of 59. State and trait anxiety scores were also very high (STAI-Y1 = 77; STAI-Y2 = 80). The Central Sensitization Inventory (CSI) score was 100/100, representing the maximum possible score and indicating a very high self-reported burden of symptoms associated with central sensitization. Overall medication adherence was assessed using the MMAS-8 and was classified as low (score = 1.25); the measure referred to the patient’s pharmacological treatment as a whole rather than specifically to galcanezumab. The PHE-S total score was 10, corresponding to a mean item score of 2 and to the “Blackout” phase of patient engagement, reflecting a low level of psychological engagement in disease management. These findings characterize the patient’s psychological status at the March 2026 assessment and should not be interpreted as longitudinal measures of treatment response.

### 3.2. Cognitive Assessment

Cognitive screening yielded a Montreal Cognitive Assessment (MoCA) raw score of 18/30, corrected to 19/30 for education (primary school level), and a Brief Cognitive Status Examination (BCSE) score of 18/58, classified as very low according to the instrument scoring framework. These results indicate reduced performance on global cognitive screening measures. Difficulties were observed across several cognitive domains, including attention, executive functioning, memory, language, and visuospatial abilities. However, given the screening nature of these instruments and the presence of relevant potential confounding factors, including severe affective symptoms, insomnia, psychiatric comorbidity, and psychotropic polypharmacotherapy, these findings should not be interpreted as establishing a specific neurocognitive disorder or a definitive multidomain/dysexecutive cognitive profile.

**Figure 2:**
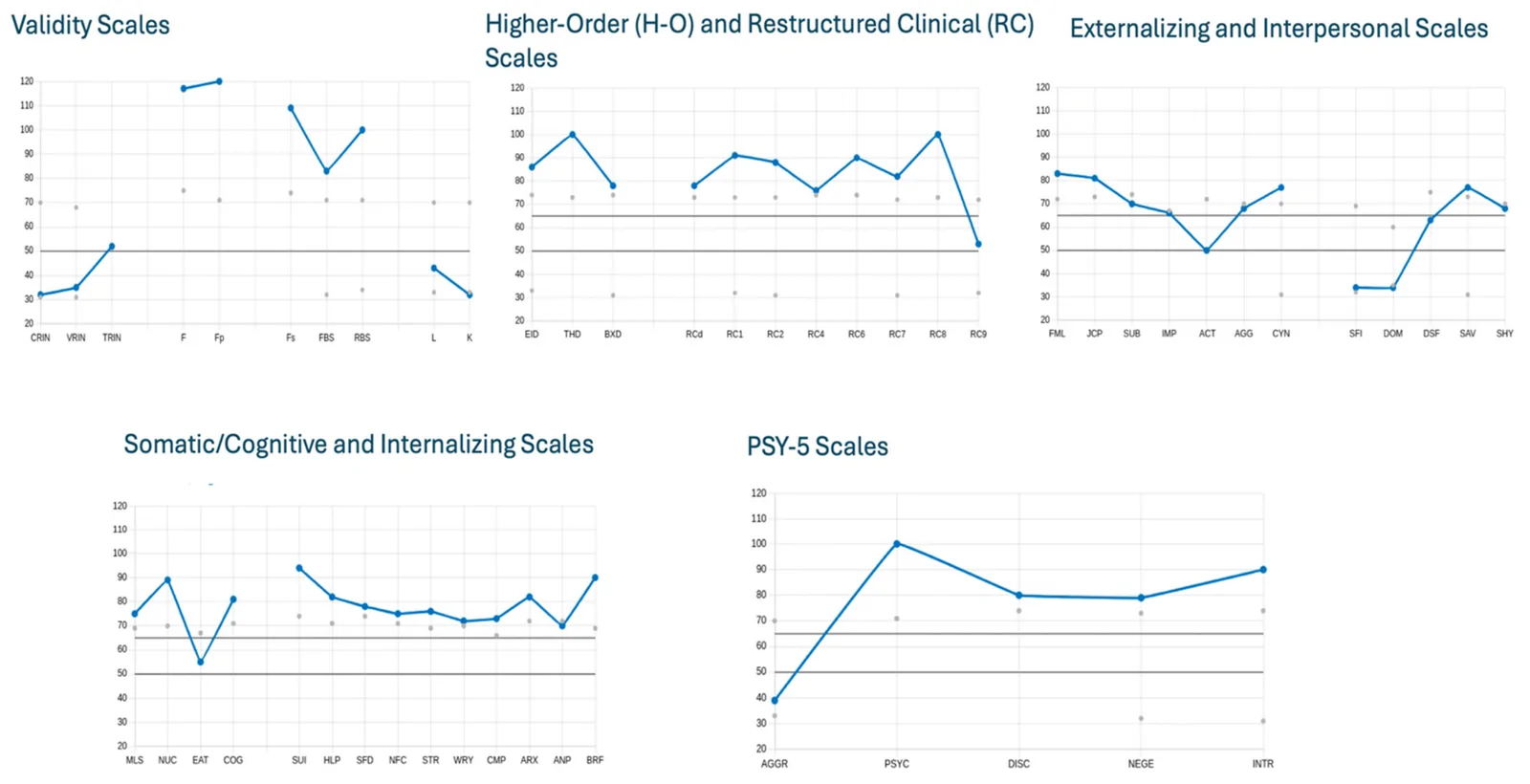
MMPI-3 profile: validity, higher-order, restructured clinical, somatic/cognitive, internalizing, externalizing, interpersonal, and PSY-5 scales.

The figure summarizes validity, higher-order, restructured clinical, somatic/cognitive, internalizing, externalizing, interpersonal, and PSY-5 scales. Validity scales showed no marked response inconsistency on CRIN, VRIN, and TRIN; however, substantial elevations were observed on infrequent-response and symptom-validity indicators (F = 117, Fp = 120, Fs = 109, FBS = 83, RBS = 100). These elevations raise concerns regarding possible symptom over-reporting and require substantial caution in the interpretation of the substantive MMPI-3 scales. Accordingly, the MMPI-3 findings were interpreted conservatively and in conjunction with the clinical interview and the broader psychological assessment, rather than as stand-alone diagnostic indicators. Substantive scale elevations were considered primarily as descriptive indicators of reported symptom burden rather than as independently valid indicators of specific psychopathological constructs. Greater interpretive weight was given to findings that were consistent with the clinical history, psychiatric follow-up, clinical interview, and broader psychological assessment. Higher-order scales showed elevations in Emotional/Internalizing Dysfunction (EID = 86), Thought Dysfunction (THD = 100), and Behavioral/Externalizing Dysfunction (BXD = 78). Restructured Clinical scales showed elevations in Somatic Complaints (RC1 = 91), Demoralization (RCd = 78), Dysfunctional Negative Emotions (RC7 = 82), Ideas of Persecution (RC6 = 90), and Aberrant Experiences (RC8 = 100). Somatic/cognitive and internalizing scales showed elevated Cognitive Complaints (COG = 81), Anxiety-Related Experiences (ARX = 82), Compulsivity (CMP = 73), Stress (STR = 76), Helplessness/Hopelessness (HLP = 82), and Suicidal/Death Ideation (SUI = 94). A clinical assessment of suicide risk was conducted during the March 2026 evaluation. No current suicidal ideation, intent, or plan emerged at the time of assessment. The patient remained under regular psychiatric follow-up. Externalizing scales showed elevations in Family Problems (FML = 83), Juvenile Conduct Problems (JCP = 81), Impulsivity (IMP = 66), and Cynicism (CYN = 77). Interpersonal scales showed elevated Social Avoidance (SAV = 77) and Shyness (SHY = 68), with low Dominance (DOM = 34). PSY-5 scales showed elevations in Psychoticism (PSYC = 100), Introversion/Low Positive Emotionality (INTR = 90), Negative Emotionality/Neuroticism (NEGE = 79), and Disconstraint (DISC = 80), with low Aggressiveness (AGGR = 39). Given the elevated SUI score and the marked depressive symptom burden, these findings were considered clinically relevant, and continued psychiatric follow-up and pharmacological management were maintained. Overall, the MMPI-3 profile showed marked elevations across several internalizing, cognitive, perceptual, and interpersonal domains; however, because of the substantial elevations in symptom-validity indicators, these findings were considered descriptive and were not interpreted diagnostically in isolation. The MMPI-3 profile, including validity, higher-order, restructured clinical, somatic/cognitive, internalizing, externalizing, interpersonal, and PSY-5 scales, is presented in Figure 2.

Overall, the MMPI-3 findings were characterized by marked elevations across internalizing, cognitive, perceptual, and interpersonal domains; however, given the substantial elevations in symptom-validity indicators, these findings should be interpreted cautiously and in conjunction with the broader clinical assessment.

## 4. Discussion

This case highlights the complexity of migraine management and treatment response in the presence of severe neuropsychiatric comorbidities.

### 4.1. “On-Off-On” Clinical Course and Response to Anti-CGRP Treatment

The observed clinical course was characterized by worsening of headache frequency following galcanezumab discontinuation and subsequent improvement after treatment reintroduction.

This “on-off-on” pattern is temporally compatible with renewed clinical benefit after galcanezumab resumption. However, because this is a single uncontrolled clinical observation, a causal relationship between treatment exposure and symptom changes cannot be established [18,19,20].

Natural fluctuations in migraine frequency, regression toward the mean, expectancy effects, changes in concomitant treatments, and variations in psychiatric or general clinical status cannot be excluded as potential contributors to the observed course [21]. Nevertheless, the temporal pattern observed in this patient is consistent with the established efficacy of galcanezumab in migraine prevention reported in randomized controlled trials and with real-world evidence supporting the effectiveness of anti-CGRP monoclonal antibodies in clinical practice [22,23,24]. 

### 4.2. Central Sensitization, Emotional Burden, and Cognitive Dysfunction

The patient’s clinical profile was characterized by a very high self-reported burden of symptoms associated with central sensitization (CSI = 100/100), associated with a severe emotional burden, including very high levels of depression and anxiety, as well as a complex psychopathological profile as reflected by the MMPI-3 assessment. From a cognitive perspective, reduced performance was observed on global cognitive screening measures, with difficulties involving attention, executive functioning, memory, language, and visuospatial abilities. However, given the screening nature of the MoCA and BCSE, these findings should not be interpreted as evidence of a definitive multidomain or dysexecutive cognitive profile. Cognitive performance should also be interpreted cautiously in light of the patient’s severe affective symptoms, insomnia, psychiatric comorbidity, and psychotropic polypharmacotherapy, all of which may have influenced test performance. The observed profile is consistent with the literature describing associations among central sensitization-related symptoms, emotional burden, and cognitive difficulties in migraine [25]. In particular, central sensitization-related symptoms have been associated with greater migraine burden and pain-related disability, while anxiety and depression represent common and clinically relevant comorbidities that may influence both pain perception and cognitive performance [26]. Furthermore, systematic reviews and meta-analyses have reported deficits in attention, memory, global cognition, and executive functions in patients with migraine, with a more pronounced impact in more complex or chronic clinical presentation [27,28,29]. However, the available evidence is not entirely consistent. Some studies have not identified marked cognitive deficits in migraine patients, suggesting that cognitive alterations may be transient or limited to specific patient subgroups [30]. Moreover, although anxiety and depression are common comorbidities, their direct impact on cognitive functioning and pain perception appears to vary across studies [31].

### 4.3. Psychiatric Comorbidity and Concomitant Treatment

The psychometric and cognitive findings obtained in March 2026 provide a cross-sectional characterization of the patient’s neuropsychiatric complexity but do not allow conclusions regarding psychiatric stabilization, treatment-mediated changes, or synergistic effects between psychotropic medications and anti-CGRP therapy. Potential interactions between psychiatric comorbidity, psychotropic treatment, central pain-related processes, and anti-CGRP response should be considered hypothesis-generating and derived from the broader literature rather than demonstrated in this patient. To date, the literature provides limited evidence specifically addressing the interaction between complex psychiatric polypharmacotherapy and response to anti-CGRP monoclonal antibodies [32,33]. Available evidence is mainly derived from observational and real-world studies of anti-CGRP treatment, which do not allow conclusions regarding specific neurobiological interactions with concomitant psychiatric treatment [34,35]. Therefore, in the present case, psychiatric comorbidity and concomitant psychotropic treatment should be interpreted as clinically relevant contextual factors rather than as mechanisms explaining the observed response to galcanezumab.

### 4.4. Discrepancy Between Treatment Adherence and Psychological Engagement

Another relevant aspect concerns the patient’s level of treatment adherence and psychological engagement. Medication adherence was classified as low according to the MMAS-8 score, while the PHE-S indicated a low level of psychological engagement in disease management. These findings suggest that both behavioral adherence and psychological involvement in care were limited at the March 2026 assessment. However, because these measures were obtained cross-sectionally, they should not be interpreted as determinants of the previous clinical response to galcanezumab. More broadly, studies in populations with chronic health conditions have shown that lower patient activation may be associated with poorer self-management and health-related outcomes [36]. However, the specific role of patient engagement in migraine remains insufficiently established, and the findings observed in the present patient should therefore be interpreted descriptively rather than as evidence of a direct relationship between engagement and headache outcomes [37].

### 4.5. Multidimensional Clinical Implications

Overall, the clinical and psychometric findings emphasize the multidimensional complexity of this case. The cross-sectional psychological and cognitive assessment provides complementary characterization of the patient’s clinical profile but cannot explain or mediate the longitudinal response to galcanezumab. Central sensitization-related symptoms, emotional distress, cognitive difficulties, and reduced patient engagement should therefore be interpreted as components of the patient’s broader clinical profile rather than as determinants of treatment response. From a clinical perspective, coordinated neurological and psychiatric monitoring may be particularly relevant in patients with migraine, severe psychiatric comorbidity, and psychotropic polypharmacotherapy.

### 4.6. Strengths and Limitations

The present case report has several strengths. First, the case is characterized by a comprehensive and multidimensional assessment integrating clinical, psychometric, cognitive, and neuropsychiatric domains, allowing for a nuanced understanding of symptomatology and treatment response. Moreover, the longitudinal description of the clinical course, particularly the “on-off-on” pattern observed in relation to the discontinuation and reintroduction of galcanezumab, represents a particularly relevant finding. Another strength lies in the contextualization of the case within a highly complex clinical setting, characterized by severe psychiatric comorbidities and polypharmacotherapy conditions frequently encountered in real-world practice but often underrepresented in controlled studies. However, several limitations should be acknowledged. First, as a case report, the findings are not generalizable and should be interpreted with caution. Additionally, the presence of multiple psychiatric comorbidities and polypharmacotherapy represents a potential confounding factor, making it difficult to isolate the specific contribution of anti-CGRP treatment. Another limitation is the lack of standardized longitudinal measurements, which prevents a systematic evaluation of the evolution of clinical, cognitive, and psychological variables over time. Finally, follow-up is still ongoing, precluding definitive conclusions regarding the long-term stability of the treatment response.

## 5. Conclusions and Future Perspectives

This case describes a clinically relevant temporal association between galcanezumab exposure and changes in headache frequency in a patient with complex neuropsychiatric comorbidity and psychotropic polypharmacotherapy. However, the single-case, uncontrolled nature of the observation precludes causal inference regarding the specific contribution of treatment exposure to the observed clinical course. The multidimensional features observed in this patient, including central sensitization-related symptoms, emotional distress, cognitive difficulties, and reduced patient engagement, should be interpreted as components of the broader clinical profile rather than as determinants of treatment response. From a clinical perspective, the case highlights the potential value of multidimensional assessment and coordinated neurological and psychiatric follow-up in patients with migraine and complex neuropsychiatric comorbidity. Future longitudinal studies with repeated psychological and cognitive assessments are needed to better characterize these complex clinical profiles.

## Figures and Tables

**Table 1:** Clinical course of migraine and galcanezumab treatment during longitudinal follow-up.

Time Point	Galcanezumab Treatment	Headache Frequency/Clinical Course	Acute Treatment	Disability Measures/Additional Information
February 2023	Initiated	Marked clinical improvement, with attack frequency reduced to approximately 1–3 attacks/month during treatment	Almotriptan, with good response	Patient followed at the Headache Center since 2018
November 2023	Continued	Approximately 3 attacks/month, rapidly responsive to acute treatment	Almotriptan	Favorable clinical course confirmed
January–February 2024	Discontinued	Approximately 4 attacks/month during the first month after discontinuation, subsequently increasing to >7 high-intensity attacks/month	Approximately 4 doses of almotriptan/month initially	Discontinuation performed in the context of clinical reassessment
March 2024	Reintroduced	Reduction to approximately 3–4 mild attacks/month after treatment resumption	Almotriptan	Clinical improvement after reintroduction
June 2024	Continued	Approximately 3–4 mild attacks/month	Almotriptan	MIDAS = 58
September 2024	Continued	Approximately 1–2 attacks/month during the preceding 3 months	Almotriptan	MIDAS = 15; HIT-6 = 48
December 2024	Continued	Approximately 1–2 attacks/month during the preceding 3 months	Almotriptan	MIDAS = 8; HIT-6 = 60
March 2025	Continued	Approximately 1–2 attacks/month during the preceding 3 months	Almotriptan, with benefit	MIDAS = 12; HIT-6 = 60
June 2025	Continued	Stable at approximately 3–4 attacks/month	Almotriptan 12.5 mg, effective	Continued preventive treatment
September 2025	Continued	Temporary increase, with peaks of 6–8 attacks/month during the summer period	Almotriptan 12.5 mg, with satisfactory response	Continued headache diary
December 2025	Continued	6–8 attacks during the preceding 30 days	Almotriptan 12.5 mg, with satisfactory response	EEG performed in December 2025
March 2026	Continued	Frequency returned to approximately 1–2 attacks/month	Almotriptan	Ongoing galcanezumab treatment

Note. Headache frequency is reported as the number of attacks per month, as documented in the available medical records. Separate monthly headache-day and migraine-day counts were not consistently available across all follow-up visits. MIDAS assesses headache-related disability over the preceding 3 months, whereas HIT-6 assesses headache impact over the preceding 4 weeks. Accordingly, MIDAS and HIT-6 scores may not directly parallel the monthly attack frequency reported at each visit. MIDAS, Migraine Disability Assessment; HIT-6, Headache Impact Test-6.

**Table 2:** Assessment domains and instruments used in the study.

Domain	Instrument	Type	Structure
Pain intensity	NPRS [9]	Clinical	Numeric rating scale, 0–10
Central sensitization-related symptoms	CSI [10]	Self-report psychometric	25-item questionnaire; total score 0–100
Cognitive function	MoCA [11], BCSE [12]	Neuropsychological	Multi-domain cognitive screening instruments
Personality and psychopathology	MMPI-3 [13]	Psychometric	335 items; standardized validity and substantive scales
Patient engagement	PHE-S [14]	Self-report psychometric	5-item ordinal scale; four engagement positions
Medication adherence	MMAS-8 [15]	Self-report behavioral	8-item medication adherence questionnaire
Psychological distress	BDI-II [16], STAI-Y1/Y2 [17]	Psychological self-report	BDI-II: 21 items; STAI-Y1/Y2: 40 items

Legend: NPRS: Numeric Pain Rating Scale; CSI: Central Sensitization Inventory; MoCA: Montreal Cognitive Assessment; BCSE: Brief Cognitive Status Examination; MMPI-3: Minnesota Multiphasic Personality Inventory-3; PHE-S: Patient Health Engagement Scale; MMAS-8: Morisky Medication Adherence Scale; BDI-II: Beck Depression Inventory-II; STAI: State-Trait Anxiety Inventory.

## Data Availability

The data that support the findings of this study are available from the corresponding author upon reasonable request.

## Abbreviations

CRIN, Combined Response Inconsistency; VRIN, Variable Response Inconsistency; TRIN, True Response Inconsistency; F, Infrequent Responses; Fp, Infrequent Psychopathological Responses; Fs, Infrequent Somatic Responses; FBS, Symptom Validity Scale; RBS, Response Bias Scale; L, Uncommon Virtues; K, Adjustment Validity; EID, Emotional/Internalizing Dysfunction; THD, Thought Dysfunction; BXD, Behavioral/Externalizing Dysfunction; RCd, Demoralization; RC1, Somatic Complaints; RC2, Low Positive Emotions; RC4, Antisocial Behavior; RC6, Ideas of Persecution; RC7, Dysfunctional Negative Emotions; RC8, Aberrant Experiences; RC9, Hypomanic Activation; MLS, Malaise; NUC, Neurological Complaints; EAT, Eating Behavior Problems; COG, Cognitive Complaints; SUI, Suicidal/Death Ideation; HLP, Helplessness/Hopelessness; SFD, Self-Doubt; NFC, Inefficacy; STR, Stress; WRY, Worry; CMP, Compulsivity; ARX, Anxiety-Related Experiences; ANP, Anger Proneness; BRF, Behavior-Restricting Fear; FML, Family Problems; JCP, Juvenile Conduct Problems; SUB, Substance Abuse; IMP, Impulsivity; ACT, Activation; AGG, Aggression; CYN, Cynicism; SFI, Self-Importance; DOM, Dominance; DSF, Disaffiliativeness; SAV, Social Avoidance; SHY, Shyness; AGGR, Aggressiveness; PSYC, Psychoticism; DISC, Disconstraint; NEGE, Negative Emotionality/Neuroticism; INTR, Introversion/Low Positive Emotionality.
